# Impact of Dexmedetomidine on Tourniquet-Induced Systemic Effects in Total Knee Arthroplasty under Spinal Anesthesia: a Prospective Randomized, Double-Blinded Study

**DOI:** 10.1155/2020/4208597

**Published:** 2020-10-05

**Authors:** Cheol Lee, Cheolhyeong Lee, Cheolhwan So, Jiheui Lee, Insung Choi, Xiao Ma, Jihyo Hwang

**Affiliations:** ^1^Department of Anesthesiology and Pain Medicine, Wonkwang University School of Medicine, 895 Muwang-ro, Iksan, Jeonllabuk-do, 54538, Republic of Korea; ^2^Department of Pediatrics, Wonkwang University School of Medicine, 895 Muwang-ro, Iksan, Jeonlabuk-do 54538, Republic of Korea; ^3^Department of Anesthesiology and Pain Medicine, Korea Cancer Center Hospital, 75 Nowon-ro, Nowon-gu, Seoul 01812, Republic of Korea; ^4^Department of Orthopaedic Surgery, Hallym University Gangnam Sacred Heart Hospital, Seoul 07441, Republic of Korea

## Abstract

**Background:**

Clinical studies on the impact of dexmedetomidine on tourniquet-induced systemic effects have been inconsistent. We investigated the impact of dexmedetomidine on tourniquet-induced systemic effects in total knee arthroplasty.

**Methods:**

Eighty patients were randomly assigned to either control (CON) or dexmedetomidine (DEX) group. The DEX group received an intravenous loading dose of 0.5 *μ*g/kg DEX over 10 minutes, followed by a continuous infusion of 0.5 *μ*g/kg/h from 10 minutes before the start of surgery until completion. The CON group received the same calculated volume of normal saline. Pain outcomes and metabolic and coagulative changes after tourniquet application and after tourniquet release were investigated.

**Results:**

The frequency of fentanyl administration postoperatively, patient-controlled analgesia (PCA) volume at 24 hours postoperatively, total PCA volume consumed in 48 hours postoperatively, and VAS score for pain at 24 and 48 hours postoperatively were significantly lower in the DEX group than in the CON group. Ten minutes after the tourniquet release, the DEX group showed significantly higher pH and lower lactate level than those in the CON group. Antithrombin III activity and body temperature 10 minutes after tourniquet release were significantly lower in the DEX group than in the CON group. Ca^2+^, K^+^, HCO_3_^−^, base excess, and PCO_2_ levels 10 minutes after tourniquet release were not significantly different between the two groups.

**Conclusion:**

We showed that DEX attenuated pain and hemodynamic, metabolic, and coagulative effects induced by the tourniquet. However, these metabolic and coagulative changes were within normal limits. Therefore, DEX could be used as an analgesic adjuvant, but should not be considered for routine use to prevent the systemic effects induced by tourniquet use.

## 1. Introduction

Tourniquet, which is widely used in surgeries of the extremities to induce a bloodless surgical field, can cause hemodynamic reaction, ischemia, and reperfusion injury, secondary to its inflation and deflation [1, 2]. The systemic effects associated with tourniquet depend on the tourniquet phase, duration of tourniquet application, size of the ischemic area, type of anesthesia administered, and the underlying cardiovascular condition of the patient [3].

The clinical findings regarding the effects of dexmedetomidine (DEX) on tourniquet-induced systemic effects have been inconsistent [4, 5]. Clinical and experimental studies focusing on the reduction of tourniquet-induced systemic effects have shown that the use of ischemic preconditioning agents, anesthetic agents, various anesthetic methods, and antioxidant agents can control tissue injury [6–10].

It has been shown that alpha-2 receptor agonists, including DEX, have a preventive effect in various ischemia-reperfusion injury models; these agonists are effective in preventing hemodynamic reactions induced by tourniquet application during surgeries of the extremities by preventing the release of catecholamines [11–13]. Few studies have reported the effects of DEX on pain outcomes and metabolic, coagulative, and temperature changes by tourniquet-induced ischemia-reperfusion injury.

We hypothesized that DEX could attenuate the systemic effects induced by tourniquet use. Therefore, we investigated the impact of DEX on pain outcomes and metabolic, coagulative, temperature, and hemodynamic changes in patients undergoing total knee arthroplasty (TKA) under spinal anesthesia.

## 2. Materials and Methods

### 2.1. Study Design

We obtained ethical approval for this study (registration no. 2020-0224) from the institutional review board in March 2020, and informed written consent was obtained from all participants. Our study was also listed on the clinicaltrials.gov (NCT04307290) on March 13, 2020.

### 2.2. Inclusion Criteria

A total of 80 patients aged between 20 and 80 years, with American Society of Anesthesiologists (ASA) physical status class I–III, who were scheduled to undergo elective TKA were enrolled in this study.

### 2.3. Exclusion Criteria

Patients with a history of rheumatoid arthritis, diabetes mellitus, hepatic or renal disease, allergy to the drug being studied, heart block greater than first degree, left ventricular ejection fraction < 55%, or chronic administration of anti-inflammatory drugs or opioids were excluded. Patients with tourniquet duration of <60 minutes or > 150 minutes or those with conversion to general anesthesia during surgery were also excluded.

### 2.4. Randomization

The randomization sequence was created using the Stata 9.0 (Stata Corp, College Station, TX, USA) statistical software and was stratified by the center with a 1 : 1 allocation using random block sizes of four. Assignments were concealed in sealed envelopes. Participants were randomly assigned following simple randomization procedures (computerized random numbers) to the DEX group (*n* = 40), which received intravenous DEX (0.5 *μ*g/kg bolus over 10 minutes, followed by 0.5 *μ*g/kg/h infusion from 10 minutes before the start of surgery to the end of surgery), or to the CON group (*n* = 40), which received an equivalent volume of normal saline bolus over 10 minutes and infusion of placebo from 10 minutes before the start of surgery until the end of the surgery.

All patients, attending anesthesiologists responsible for patient care, and nurses were blinded to the anesthetic agent during the study period.

### 2.5. Anesthesia and Perioperative Care

Standard monitoring included electrocardiography, noninvasive arterial blood pressure monitoring, and pulse oximetry. All patients received spinal anesthesia administered by an attending anesthesiologist, and 500 mL Ringer's lactate solution was administered to ensure hydration before spinal anesthesia.

Spinal anesthesia was induced in the lateral decubitus position. These patients received an intrathecal injection of 15 mg 0.5% bupivacaine (in 5% glucose) at the level of L4-5 via a 25-gauge needle. The patients were then turned to the supine position, and the level of sensory block was evaluated by a pinprick.

The peak level of sensory block, the sensory dermatome at tourniquet pain, the time between tourniquet application and the onset of pain, and the tourniquet pain were determined by an independent researcher at 5, 10, 15, and 20 minutes after the spinal injection and then at 10-minute intervals until the complete resolution of the sensory block. The affected extremity in all patients was exsanguinated with an Esmarch bandage, and a tourniquet was applied at a pressure of 300 mmHg during the surgery.

When the patient felt a poorly localized and diffuse pain after tourniquet inflation, despite adequate sensory blockade during surgery, the pain was considered to be induced by the tourniquet. Intravenous fentanyl was administered at a dose of 100 *μ*g as supplemental analgesia if the patients experienced tourniquet pain at any time during the procedure. General anesthesia was induced if intravenous fentanyl was ineffective. The need for supplementary intravenous fentanyl and any conversion to general anesthesia were recorded. In case of intraoperative bleeding, volume replacement with Ringer's lactate solution was performed, according to the decision of an attending anesthesiologist instead of blood products.

Systolic blood pressure (SBP), diastolic blood pressure (DBP), mean arterial blood pressure (MAP), and heart rate (HR) were monitored every 5 minutes until the end of surgery. Hypotension was defined as a 30% reduction in basal MAP, which was treated with 5 mg intravenous ephedrine administration. When the HR was <50 beats/min (bradycardia), 0.2 mg glycopyrrolate was injected intravenously.

Before the start of surgery, a femoral nerve block was performed to reduce postoperative pain using a bolus of 20 mL 0.75% ropivacaine. Concurrently, patient-controlled analgesia (PCA) pump containing 1000 *μ*g fentanyl, 150 mg ketorolac, and 0.6 mg ramosetron in 150 mL saline was set to deliver a basal infusion of 2 mL/h and bolus doses of 1 mL, with a 15-minute lockout period for postoperative analgesia. The PCA volume consumed at 24 and 48 hours after surgery and total PCA volume consumed in 48 hours after surgery were recorded. Postoperative pain intensity at rest was measured using a 100 mm linear visual analog scale (VAS). The VAS score for pain at rest was measured at 24 and 48 hours after surgery. Fentanyl in a dose of 100 *μ*g was administered for pain with VAS score ≥ 50, or 30 mg ketorolac was administered for pain with VAS score < 50 or on patient request.

### 2.6. Measurements of Metabolic, Coagulative, and Temperature Changes

Arterial blood samples were obtained from the radial artery 1 minute before the start of the spinal anesthesia as a baseline value and at 10 minutes after tourniquet release.

Five milliliters of blood samples were extracted through the radial artery, and 1 mL was collected in a heparinized syringe; following this, the remaining blood samples were immediately sent through the pneumatic tube system, guaranteeing their processing in the least amount of time possible, not >5 minutes by institutional standards. The metabolic variables included pH, lactate, calcium, potassium, bicarbonate, CO_2_, and base excess, and coagulative change included antithrombin III activity.

Tympanic temperature measurements were made using the Braun Thermoscan (Thermoscan Inc., San Diego, CA) inserted into the auditory canal at 1 minute just before the start of spinal anesthesia as a baseline and at 10 minutes after tourniquet release.

### 2.7. Statistical Analysis

The sample size was calculated using the PASS 2008 (NCSS, LLC. Kaysville, Utah, USA) software. A preliminary investigation had shown that the mean ± standard deviation (SD) of the two treatment groups for total PCA volume used in 48 hours after surgery as a primary outcome was 125.67 ± 8.2 and 120.90 ± 5.8, respectively. The investigation revealed that a sample size of 36 patients per group would enable the detection of a significant difference with a power of 80% and an *α*-coefficient of 0.05. The final sample size of this study was determined to be 40 patients per group, with a dropout rate of 10%. The SPSS version 18.0 (SPSS Inc., Chicago, IL, USA) was used for the statistical analysis. The data are presented as mean ± SD, median (interquartile range), or number (%) of patients. The groups were compared using an independent *t*-test or the Mann–Whiney *U* test according to normality test and categorical variables with the *χ*^2^ test or Fisher exact test as appropriate.

The group-by-time interaction assesses whether the change over time differs between groups. Repeated measure analysis of variance was used for intragroup comparisons with *P* values that were adjusted with the Bonferroni correction for multiple comparisons. A *P* value <0.05 was considered statistically significant.

## 3. Results

**Figure 1:**
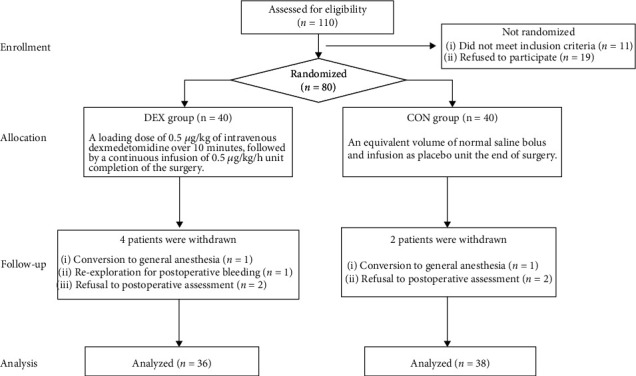
Consort flow diagram.

A total of 110 patients were assessed for eligibility, and 30 were excluded, because 11 did not meet the inclusion criteria and 19 refused to participate. Eighty patients received study medication after randomization. Six patients were initially enrolled but were withdrawn due to conversion to general anesthesia, re-exploration for postoperative bleeding, or refusal for postoperative assessment (Figure 1).

Age, sex, weight, height, ASA classification, and duration of surgery were not significant between the two groups (Table 1). Tourniquet time, tourniquet pain, amount of intraoperative fentanyl administration, the time between tourniquet application and the onset of pain, the peak level of sensory block, sensory dermatome at tourniquet pain, intraoperative blood loss, intravenous fluid administered, hypotension, postoperative delirium, amount of postoperative ketorolac administration, and PCA volume consumed at 48 hours after surgery were not significantly different between the two groups. The DEX group had significantly lower postanesthetic shivering (*P* = 0.025); the frequency of fentanyl administration postoperatively (*P* = 0.013), PCA volume at 24 hours after surgery (*P* < 0.01), total PCA volume consumed in 48 hours after surgery (*P* < 0.01), and VAS score for pain at 24 hours (*P* < 0.01) and 48 hours after surgery (*P* < 0.01) were also significantly lower in the DEX group than the CON group (Table 2).

At 10 minutes after tourniquet release, the pH of arterial blood was significantly higher (*P* < 0.022) and lactate was significantly lower (*P* < 0.014) in the DEX group compared with the CON group. The antithrombin III activity (*P* < 0.01) and body temperature (*P* < 0.012) at 10 minutes after tourniquet release in the DEX group were significantly lower than in the CON group. Ca2^+^, K^+^, HCO_3_^−^, base excess, and PCO_2_ at 10 minutes after tourniquet release were not significantly different between the two groups (*P* > 0.05) (Table 3).

SBP, DBP, MAP, and HR at 10 minutes after tourniquet application were not significantly different between the two groups. SBP, DBP, and MAP at 30 minutes after tourniquet application were not significantly different between the two groups, but HR was lower in the DEX group than in the CON group (*P* < 0.01). SBP, DBP, MAP, and HR at 60 minutes after tourniquet application and at 10 minutes after tourniquet release were lower in the DEX group than that in the CON group (*P* < 0.05) (Table 4).

pH, lactate, Ca^2+^, K^+^, base excess, PCO_2_, antithrombin III activity, and body temperature at 10 minutes after tourniquet release were significantly changed (P < 0.01) when compared to baseline values (Table 5).

SBP, DBP, MAP, and HR at 10, 30, and 60 minutes after tourniquet application and at 10 minutes after tourniquet release were significantly decreased when compared to baseline values. SBP, DBP, MAP, and HR at 60 minutes after tourniquet application were significantly higher than that at 30 minutes after tourniquet application. SBP, DBP, MAP, and HR at 10 minutes after tourniquet release were significantly lower than that at 60 minutes after tourniquet application. (P < 0.01) (Table 6).

## 4. Discussion

The main findings of our study demonstrated that DEX reduced the frequency of postoperative fentanyl administration, PCA volume consumed after surgery, and VAS score for pain after surgery compared to saline. At 10 minutes after tourniquet release, the pH of arterial blood was significantly higher and the lactate level was significantly lower in the DEX group than in the CON group. The antithrombin III activity and body temperature at 10 minutes after tourniquet release were significantly lower in the DEX group than in the CON group. Ca^2+^, K^+^, HCO_3_^−^, base excess, and PCO_2_ levels at 10 minutes after tourniquet release were not significantly different between the two groups. DEX attenuated pain and hemodynamic, metabolic, and coagulative effects induced by tourniquet use. However, these metabolic and coagulative changes were within normal limits. Dexmedetomidine is a potent alpha-2-adrenergic agonist, more selective than clonidine, with widespread actions on the mammalian brain.

Several systemic effects, which are temporary but can be dramatic, occur with tourniquet inflation and deflation during surgeries of the extremities. These systemic effects might cause hemodynamic, central nervous system, hematologic, temperature, and metabolic changes. Several studies have been performed on the effects of the patient's cardiovascular condition, various anesthetic agents, and anesthetic methods on these systemic effects induced by tourniquet-induced ischemia-reperfusion injury [3–5]. One anesthetic technique was selected, the recommended dosage of dexmedetomidine was used, and tourniquet time was usually over 60 minutes for reducing the bias. DEX reduces postoperative analgesic (fentanyl) administration, VAS pain scores at 24 and 48 hours after surgery, PCA volume consumed at 24 hours, and total PCA volume consumed in 48 hours after surgery.

Tourniquet-induced hemodynamic changes occur according to the tourniquet phases. A gradual increase in arterial pressure is frequently observed after tourniquet inflation, which increases blood volume and systemic vascular resistance. A sudden drop in the central venous pressure and mean arterial pressures after tourniquet deflation is due to the combination of a shift in blood volume back into the limb and washout of metabolites from the ischemic limb into the systemic circulation. However, these changes are minimal in healthy patients, although patients with poor cardiac function may not be able to tolerate these changes [2, 3]. Hemodynamic changes according to tourniquet use were within normal limits, although DEX in this study attenuated hemodynamic responses induced by tourniquet use when compared to saline.

The tourniquet-induced systemic changes except for postoperative pain outcomes in this study were transient but minimal, although DEX reduced these changes. These results might be due to the study design (spinal anesthesia and good cardiovascular conditions with EF ≥ 55%). Koşucu et al. [14] reported that total intravenous anesthesia with propofol could be better compared to inhalational anesthesia or spinal anesthesia, which were not significant. Their study with a shorter duration of tourniquet application with 64–68 minutes is different from our study with 92–93 minutes of tourniquet application.

Previous studies have reported on the effects of DEX on ischemia-reperfusion injury induced by tourniquet use and focused on serum levels of proinflammatory cytokines, malondialdehyde, a biomarker of oxidative stress or hemodynamic changes [4, 5]. This study reported that DEX reduced pain outcomes and metabolic, coagulative, temperature, and hemodynamic changes by tourniquet-induced ischemia-reperfusion injury.

Tourniquet pain is characterized as a dull, aching pain occurring after spinal or epidural anesthesia, despite adequate anesthesia of the sensory dermatome underlying the tourniquet. Various interventions using adjuncts to bupivacaine such as epinephrine, clonidine and morphine, gabapentin, or ketamine have been used to prevent tourniquet pain [2]. Tourniquet pain in this study occurred in 16.7% of patients in the CON group and 7.9% in the DEX group, and fentanyl was administered in 11.1% of patients in the CON group and 5.3% in the DEX group. DEX decreased the frequency of tourniquet pain and fentanyl administration when compared to saline, but the difference was not significant; however, DEX did reduce pain outcomes as shown by VAS pain score intensity and PCA volume consumed after surgery. These results might be due to the relatively high density of bupivacaine (15 mg) or the small sample size. Administration of 15 mg hyperbaric bupivacaine to the two groups may have masked the analgesic effect of DEX on tourniquet pain.

Postanesthetic shivering may be thermoregulatory in response to core hypothermia or due to cytokine release by the surgical procedure or nonthermoregulatory in relation to certain anesthetics or postoperative pain. Alpha2 receptor agonists including DEX are an important class of antishivering drugs and reduce both the vasoconstriction and shivering thresholds [15, 16]. This study showed that the DEX group had significantly lower body temperature and lower incidence of shivering than the CON group. However, several studies reported that certain patients with DEX used for sedation in the intensive care unit developed high temperatures without clear alternative causes [17, 18]. DEX-associated hyperthermia mechanisms are currently unknown. Because the temperature environment, patient conditions, or intervention applied between the operating room and intensive care unit are different, further studies are required to clarify the effect of DEX on body temperature.

There were some limitations to this study. First, we took two blood samples from patients sequentially. Our institution review board recommended a maximum of two chances to draw blood due to economic and ethical issues. The arterial blood samples were obtained from the radial artery 1 minute before the start of spinal anesthesia as a baseline and at 10 minutes after tourniquet release. It was difficult to evaluate metabolic and coagulative changes induced by the tourniquet phases sequentially. Second, although the sample size was determined by the total PCA volume consumed in 48 hours after surgery as a primary outcome, the sample size in this study may have been inadequate to evaluate the detailed metabolic and coagulative changes. Small sample size may mislead the researcher when it comes to making clinical decisions. Further, a larger sample size may be needed to clarify the effect of DEX on metabolic and coagulative changes induced by tourniquet application.

## 5. Conclusions

In conclusion, we report that DEX attenuated pain and hemodynamic, metabolic, and coagulative effects induced by tourniquet use. However, these hemodynamic, metabolic, and coagulative changes were within normal limits. The clinical importance of this small effect is questionable. Nevertheless, the conclusion on DEX and metabolic effects may not be valid, since the biomarkers chosen for metabolic effects are probably not sensitive enough to reveal the effect of DEX on tourniquet-induced reperfusion injuries.

## Figures and Tables

**Table 1:** Patients' characteristics.

	CON group (*n* = 36)	DEX group (*n* = 38)	*P* value
Age (years)	76.2 ± 7.9	75.5 ± 9.6	0.75
Sex (M/F)	13/23	15/23	0.77
Weight (kg)	65.8 ± 8.3	66.4 ± 8.0	0.76
Height (cm)	157.6 ± 9.6	158.6 ± 9.6	0.66
ASA (I/II/III)	0/19/17	0/20/18	0.99
Duration of surgery (minutes)	115.0 ± 9.5	120.5 ± 18.8	0.84
Duration of anesthesia (minutes)	140.6 ± 14.7	141.3 ± 17.3	0.12

Values are expressed as mean ± SD or number of patients.

**Table 2:** Perioperative data.

	CON group (*n* = 36)	DEX group (*n* = 38)	*P* value
Tourniquet time (minutes)	93.5 ± 7.6	92.0 ± 6.1	0.35
Tourniquet pain	6 (16.7)	3 (7.9)	0.25
The frequency of fentanyl administered intraoperatively (100 *μ*g)	4 (11.1)	2 (5.3)	0.36
Time between tourniquet application and onset of pain (min)	69.2 ± 5.8	78.3 ± 2.9	0.33
Peak level of sensory block	T9 (T7, T10)	T 9 (T7, T10)	0.13
Sensory dermatome at tourniquet pain	T10 (T10, T12)	T10 (T9, T11)	0.41
Intraoperative blood loss (mL)	366.5 ± 22.4	350.9 ± 12.1	0.35
Intravenous fluid administered (mL)	1550.9 ± 280.7	1640.5 ± 330.4	0.21
Hypotension	6 (16.7)	10 (26.3)	>0.05
Bradycardia	6 (16.7)	16 (42.1)	0.017
Postoperative delirium	3 (8.3)	1 (2.6)	>0.05
Shivering	10 (27.8)	3 (7.9)	0.025
Additional analgesic type administered postoperatively	31 (86.1)	23 (60.5)	0.013
Ketorolac	12 (33.3)	12 (31.6)	>0.05
Fentanyl	19 (52.8)	11 (28.9)	0.037
PCA volume consumed at 24 hours after surgery (mL) at rest	73.28 ± 4.44	56.42 ± 4.56	0.00
PCA volume consumed at 48 hours after surgery (mL) at rest	53.50 ± 4.09	52.42 ± 4.79	0.30
Total PCA volume consumed for 48 hours after surgery (mL)	126.78 ± 4.91	108.84 ± 8.59	0.00
VAS for pain at 24 hours after surgery	60.56 ± 7.15	46.05 ± 9.17	0.00
VAS for pain at 48 hours after surgery	41.94 ± 5.25	31.32 ± 4.75	0.00

Values are expressed as mean ± SD, median (interquartile range) or number (%) of patients;

Abbreviation: PCA: patient-controlled analgesia; VAS: visual analogue scale; CON: control; DEX: dexmedetomidine; SD: standard deviation.

**Table 3:** Metabolic, coagulative, and temperature changes in arterial blood at 10 minutes after tourniquet release between groups.

	CON (*n* = 36)	DEX (*n* = 38)	*P* value
pH at baseline	7.41 ± 0.02	7.41 ± 0.02	0.14
pH at 10 minutes after tourniquet release	7.38 ± 0.03	7.38 ± 0.02	0.02
Lactate at baseline (Mm/L)	0.83 ± 0.21	0.89 ± 0.18	0.17
Lactate at 10 minutes after tourniquet release (Mm/L)	1.89 ± 0.41	1.65 ± 0.39	0.01
Ca^2+^ at baseline (mg/dL)	1.16 ± 0.01	1.15 ± 0.03	0.51
Ca^2+^ at 10 minutes after tourniquet release (mg/dL)	1.18 ± 0.04	1.18 ± 0.04	0.59
K^+^ at baseline (mEq/L)	3.89 ± 0.19	3.81 ± 0.47	0.38
K^+^ at 10 minutes after tourniquet release (mEq/L)	4.22 ± 0.16	4.14 ± 0.51	0.42
HCO_3_^−^ at baseline (Mm/L)	23.31 ± 2.37	23.76 ± 2.12	0.39
HCO_3_^−^ at 10 minutes after tourniquet release (Mm/L)	23.83 ± 1.68	23.56 ± 1.33	0.45
PCO_2_ at baseline (mmHg)	38.47 ± 2.71	39.66 ± 3.51	0.11
PCO_2_ at 10 minutes after tourniquet release (mmHg)	41.00 ± 3.00	40.71 ± 2.61	0.55
Base excess at baseline	0.07 ± 1.99	0.22 ± 1.87	0.73
Base excess at 10 minutes after tourniquet release	-0.82 ± 1.85	−0.92 ± 1.74	0.82
Antithrombin III activity (%) at baseline	94.50 ± 8.71	95.89 ± 7.85	0.13
Antithrombin III activity (%) at 10 minutes after tourniquet release	84.47 ± 5.53	82.68 ± 6.42	0.021
Body temperature at baseline (°C)	36.7 ± 0.2	36.7 ± 0.3	0.67
Body temperature at 10 minutes after tourniquet release (°C)	36.2 ± 0.4	36.0 ± 0.2	0.012

Values are expressed as mean ± SD or number (%) of patients. Abbreviation: CON: control; DEX: dexmedetomidine; SD: standard deviation.

**Table 4:** Hemodynamic changes after tourniquet application and release between the two groups.

	CON (*n* = 36)	DEX (*n* = 38)	*P* value
Baseline			
SBP (mmHg)	143.1 ± 9.0	146.0 ± 9.0	0.16
DBP (mmHg)	85.3 ± 10.4	85.5 ± 9.8	0.93
MAP (mmHg)	104.4 ± 9.1	107.9 ± 8.0	0.83
HR (beats/min)	63.8 ± 11.3	61.4 ± 6.4	0.27
10 minutes after tourniquet application			
SBP (mmHg)	125.9 ± 9.4	124.2 ± 6.5	0.36
DBP (mmHg)	77.9 ± 8.7	76.6 ± 6.2	0.47
MAP (mmHg)	92.6 ± 9.6	91.4 ± 9.9	0.61
HR (beats/min)	58.5 ± 4.2	53.0 ± 9.3	0.00
30 minutes after tourniquet application			
SBP (mmHg)	117.0 ± 1 1.5	112.5 ± 10.1	0.08
DBP (mmHg)	72.3 ± 10.1	68.5 ± 10.0	0.10
MAP (mmHg)	87.2 ± 10.4	83.1 ± 9.8	0.08
HR (beats/min)	53.8 ± 4.2	51.2 ± 3.3	0.00
60 minutes after tourniquet application			
SBP (mmHg)	128.3 ± 7.1	122.9 ± 7.9	0.00
DBP (mmHg)	81.2 ± 9.8	74.2 ± 11.6	0.00
MAP (mmHg)	96.9 ± 8.4	90.5 ± 10.1	0.00
HR (beats/min)	57.4 ± 4.2	54.2 ± 7.1	0.025
10 minutes after tourniquet release			
SBP (mmHg)	109.6 ± 11.2	104.4 ± 6.0	0.01
DBP (mmHg)	68.7 ± 10.2	62.6 ± 4.2	0.00
MAP (mmHg)	82.4 ± 10.0	76.6 ± 4.6	0.00
HR (beats/min)	63.5 ± 11.1	55.9 ± 5.5	0.00

Values are expressed as mean ± SD or number (%) of patients. Abbreviation: SBP: systolic blood pressure; DBP: diastolic blood pressure; MAP: mean arterial blood pressure; HR: heart rate; SD: standard deviation; CON: control; DEX: dexmedetomidine.

**Table 5:** Metabolic, coagulative, and temperature changes in arterial blood at 10 minutes after tourniquet release.

	Baseline	Tourniquet release	*P* value
pH	7.41 ± 0.17	7.38 ± 0.03	0.00
Lactate (Mm/L)	0.86 ± 0.20	1.77 ± 0.41	0.00
Ca2^+^ (mg/dL)	1.16 ± 0.02	1.50 ± 1.11	0.00
K^+^(mEq/L)	3.85 ± 0.36	4.17 ± 0.38	0.00
HCO_3_^−^ (Mm/L)	23.54 ± 2.24	23.65 ± 1.50	0.28
PCO_2_ (mmHg)	38.46 ± 5.42	40.30 ± 4.7	0.00
Base excess	0.15 ± 1.91	−0.87 ± 1.78	0.00
Antithrombin III activity (%)	91.72 ± 7.60	83.44 ± 7.05	0.00
Body temperature (°C)	36.7 ± 0.2	36.1 ± 0.3	0.00

**Table 6:** Hemodynamic changes after tourniquet application and release.

	Baseline	10 min after tourniquet application	30 min after tourniquet application	60 min after tourniquet application	10 min after tourniquet release
SBP (mmHg)	144.6 ± 9.1	125.1 ± 8.0^∗^*^ΙΙ^*	114.7 ± 11.0^∗^^†*ΙΙ*^	125.6 ± 8.0^∗^^‡*ΙΙ*^	107.0 ± 9.2^∗^^§^
DBP (mmHg)	85.4 ± 10.1	77.2 ± 7.5^∗^*^ΙΙ^*	70.3 ± 10.2^∗^^†*ΙΙ*^	77.6 ± 11.2^∗^^‡*ΙΙ*^	65.6 ± 8.3^∗^^§^
MAP (mmHg)	106.2 ± 8.7	92.0 ± 9.7^∗^*^ΙΙ^*	85.1 ± 10.3^∗^^†*ΙΙ*^	93.6 ± 9.8^∗^^‡*ΙΙ*^	79.4 ± 8.2^∗^^§^
HR (beats/min)	62.5 ± 9.1	55.7 ± 7.8^∗^*^ΙΙ^*	52.5 ± 3.9^∗^^†*ΙΙ*^	55.8 ± 6.1^∗^^‡*ΙΙ*^	59.6 ± 9.4^∗^^§^

^∗^ *P* < 0.01 vs. baseline, ^†^*P* < 0.01 vs. 10 min after tourniquet application, ^‡^*P* < 0.01 vs. 30 min after tourniquet application, ^§^*P* < 0.01 vs. 60 min after tourniquet application. Abbreviation: SBP: systolic blood pressure; DBP: diastolic blood pressure; MAP: mean arterial blood pressure; HR: heart rate.

## Data Availability

The data used to support the findings of this study are included in the article. We can share our data; our data is also available.
